# Model of Ovarian Adenocarcinoma Spheroids for Assessing Photodynamic Cytotoxicity

**DOI:** 10.17691/stm2020.12.1.04

**Published:** 2020

**Authors:** E.A. Sokolova, A.O. Senatskaya, S.A. Lermontova, E.K. Akinchits, L.G. Klapshina, A.A. Brilkina, I.V. Balalaeva

**Affiliations:** Junior Researcher, Laboratory of Optical Theranostics, Institute of Biology and Biomedicine, National Research Lobachevsky State University of Nizhni Novgorod, 23 Prospekt Gagarina, Nizhny Novgorod, 603950, Russia; Graduate Student, Institute of Biology and Biomedicine, National Research Lobachevsky State University of Nizhni Novgorod, 23 Prospekt Gagarina, Nizhny Novgorod, 603950, Russia; Researcher, Laboratory for Search and Applied Research, G.A. Razuvaev Institute of Organometallic Chemistry, Russian Academy of Sciences, 49 Tropinina St., Nizhny Novgorod, 603137, Russia; Researcher, Department of Biophysics, Institute of Biology and Biomedicine, National Research Lobachevsky State University of Nizhni Novgorod, 23 Prospekt Gagarina, Nizhny Novgorod, 603950, Russia; Senior Researcher, Sector of Chromophore Compounds for Medicine, G.A. Razuvaev Institute of Organometallic Chemistry, Russian Academy of Sciences, 49 Tropinina St., Nizhny Novgorod, 603137, Russia; Associate Professor, Senior Researcher, Laboratory of Optical Theranostics, Institute of Biology and Biomedicine, National Research Lobachevsky State University of Nizhni Novgorod, 23 Prospekt Gagarina, Nizhny Novgorod, 603950, Russia; Associate Professor, Senior Researcher, Laboratory of Optical Theranostics, Institute of Biology and Biomedicine, National Research Lobachevsky State University of Nizhni Novgorod, 23 Prospekt Gagarina, Nizhny Novgorod, 603950, Russia

**Keywords:** photodynamic therapy, photosensitizer, tumor spheroid, human ovarian adenocarcinoma, porphyrazines

## Abstract

**The aim of the study** was to compare the relevance of ovarian adenocarcinoma spheroids with that of a monolayer culture for assessing photodynamic effect of the tetrakis(4-benzyloxyphenyl)tetracyanoporphyrazine photosensitizer.

**Materials and Methods:**

The work was performed on SKOV-3 human ovary adenocarcinoma cells grown *in vitro* in a monolayer culture and in the form of tumor spheroids obtained using culture plates with ultra-low attachment. We determined the photoinduced toxicity of porphyrazine on a monolayer culture using the MTT assay; the effect on the spheroids was tested by assessing the dynamics of their growth. Cellular uptake of porphyrazine was analyzed by confocal microscopy.

**Results:**

Porphyrazine has a pronounced photodynamic effect on SKOV-3 cells. When exposed to light at a dose of 20 J/cm^2^, the IC_50_ value 24 h after exposure was 2.3 μM for SKOV-3 monolayer culture. For the spheroids, the effect manifested after a latency period: significant growth retardation of the treated spheroids appeared no sooner than 5 and 9 days after exposure. Notably, no decrease in the initial size of the treated spheroids was observed under any of the photodynamic regimes. The penetration depth of porphyrazine into spheroids was 50–100 μm during 24 h incubation.

**Conclusion:**

The limited penetration of the photosensitizer into the body of spheroids and its predominant accumulation in the surface layers can be one of the key factors behind the significant differences in the photodynamic response between the surface and deep layers of a spheroid. For cells located close to the spheroid surface, the photodynamic effect is comparable to that for a monolayer culture, while in deeper layers, the cells remain viable and support/maintain the growth of the spheroid even under intense photo-exposure. The fact that the *in vitro* distribution is similar to the inhomogeneous accumulation of photosensitizers in tumors *in vivo* allows us to consider spheroids more relevant than a monolayer culture for studying photodynamic anti-tumor effects.

## Introduction

Photodynamic therapy (PDT) is a minimally invasive, rapidly developing method for the diagnosis and treatment of malignant neoplasms and non-malignant diseases. A photodynamic effect is induced by irradiating a photoactive dye (photosensitizer) with monochromic light in the presence of oxygen [1, 2]. As a result, reactive oxygen species (ROS) are produced, which triggers a chain free radical processes that cause damage to biomolecules and membranes. Ultimately, signaling pathways in the cell are activated, leading to its death [3]. Singlet oxygen is considered to be the main effector molecule in PDT [4], but other ROS, such as hydrogen peroxide [5], superoxide anion radical, and hydroxyl radical [6] play a significant role.

Chemical compounds used as photosensitizers can be classified into porphyrins and non-porphyrins [7]. Of non-porphyrin compounds, those based on phenothiazine dyes (e.g., methylene blue, toluidine blue, and Nile blue) and cyanines, as well as polycyclic aromatic compounds (e.g., hypericin) have been used most often. Photoactive dyes with the tetrapyrrole structure include hematoporphyrins, protoporphyrin XI and the second-generation drugs: porphyrins, chlorins and bacteriochlorins, pheophorbides, purpurins, protoporphyrins, porphyrazines, phthalocyanines, etc. [8–12].

Compounds of the tetra(aryl)tetracyanoporphyrazines family are considered promising agents for PDT [13, 14]. A unique feature of this group of photosensitizers is the high sensitivity of their photophysical parameters to viscosity. This behavior is due to the belonging of these compounds to the so-called fluorescent molecular rotors. The porphyrazines of the named group are characterized by intramolecular rotation of side radicals of the macrocycle upon photon absorbing, which ensures non-radiative relaxation of the excited state of the molecule [15]. An increasing viscosity of the medium interferes with the radicals rotation. This is manifested in a multiple increase in the quantum yield and fluorescence lifetime. Earlier [16], we showed the possibility of using compounds of this group for monitoring intracellular viscosity during cell response to PDT. In the future, the approach based on the use of the photosensitizers with rotor properties can become the basis for the development of PDT methods with real-time monitoring of the functional state of the irradiated tissue.

Traditionally, new photoactive compounds are tested using monolayer cell cultures. Such cultures have a number of undoubted advantages: they are easily produced and maintained, and they enable the investigator to monitor individual cells in the culture. However, two-dimension monolayer cultures do not reflect some significant features of real tumors because the latter have a three-dimensional structure. For example, a tumor is characterized by gradients of gases, nutrients, and catabolites, as well as the presence of non-tumorous cells and extracellular matrix. This specific microenvironment determines the heterogeneity of a tumor in terms of metabolism, gene expression and resistance to therapeutics [17, 18].

Due to the above circumstances, three-dimensional (3D) *in vitro* models of tumors are becoming more common: from spherical conglomerates of tumor cells (spheroids) to complex multicomponent models that include (in addition to tumor cells) cellular components of the tumor stroma as well as the extracellular matrix [19, 20]. The structural similarity of 3D models to real tumors implies their higher relevance compared to monolayer cultures. To date, a number of studies have been published that show the importance of 3D structure *in vitro* in the molecular processes triggered in lung and colon carcinomas in response to photodynamic treatment and, as a consequence, in the sensitivity of cells to PDT [21, 22]. No such studies pertaining to ovarian adenocarcinoma have been reported.

**The aim of the study** was to compare the relevance of ovarian adenocarcinoma spheroids with that of a monolayer culture for assessing photodynamic effects of the tetrakis(4-benzyloxyphenyl)tetracyanoporphyrazine photosensitizer.

## Materials and Methods

***Preparation of human ovarian adenocarcinoma SKOV-3 spheroids.*** In this study, we used a cell line of human ovarian adenocarcinoma SKOV-3 (ATCC catalog number HTB-77). Cells were cultured in a CO_2_ incubator (5% CO_2_, 37°C) using the McCoy’s 5A growth medium containing 1.5 mM glutamine (HyClone, USA) with the addition of 10% fetal calf serum (HyClone). Versen’s solution (PanEco, Russia) was used to detach cells from the substrate.

To obtain spheroids, a suspension of SKOV-3 cells was plated on 96-well round-bottom plates with ultra-low attachment (Ultra-Low Attachment Microplate; Corning, USA) at 500 cells per well and cultured for 3 days until well-shaped cell conglomerates were formed [23]. During the long-term cultivation of spheroids, the medium was replaced every 7 days to maintain cell viability.

***Viability of SKOV-3 cells in a monolayer after photodynamic treatment.*** We used a novel photoactive compound of the family of porphyrazines — tetrakis(4-benzyloxyphenyl)tetracyanoporphyrazine (hereinafter, porphyrazine), synthesized at the G.A. Razuvaev Institute of Organometallic Chemistry, Russian Academy of Sciences (Nizhny Novgorod) [24].

SKOV-3 cells were plated on two 96-well culture plates at 2000 cells per well. In 24 h, the growth medium was replaced with serum-free McCoy’s 5A medium containing porphyrazine at 0.1 to 10 μM; the cells were further incubated for 4 h in a CO_2_ incubator. Then, the porphyrazine containing medium was replaced with fresh serum containing medium. After that, one of the plates was irradiated with a LED-light source at a wavelength of 615–635 nm and a dose of 20 J/cm^2^ [25]. At this time, the second culture plate (control) was kept in the dark. Then, the cells in both plates were incubated for 24 h and their viability was estimated using the MTT assay [26]. To that end, the medium was replaced with fresh medium containing MTT at 0.5 mg/ml (Alfa Aesar, Great Britain) and incubated for another 4 h. Then, formazan crystals formed from MTT were dissolved in dimethyl sulfoxide (PanEco); the optical density of the resulting samples was measured at λ=570 nm using a Synergy MX plate spectrophotometer (BioTek, USA). The relative cell viability was presented as the ratio of optical densities of treated and untreated samples. Based on the obtained data, the IC_50_ value of porphyrazine was calculated by nonlinear regression using the four-parameter dose-effect model in GraphPad Prism 6.0 (GraphPad Software).

***Growth of SKOV-3 spheroids after photodynamic treatment.*** Spheroids SKOV-3 were incubated in serum-free McCoy’s 5A medium containing porphyrazine in the same concentrations as for the monolayer. After 4 h, the medium was replaced with serum-supplemented medium; then, the photodynamic treatment was performed under the same conditions as for the monolayer culture. After the exposure, the spheroid cultures were maintained in a CO_2_ incubator for 9 days.

Images of spheroids were obtained daily by using an Axiovert 200 inverted microscope and an EC Plan-Neofluar 10×/0.3 M27 objective lens (Carl Zeiss, Germany). Microscope AxioVision LE software was used to determine the size of spheroids. Spheroid volume (*V*, μm^3^) was calculated according to equation *V*=*a*·*b*^2^/2, where *a* is the larger diameter (μm) and *b* is the smaller diameter (μm). The volume of each spheroid at each time point was presented as percent of the volume on the day of treatment. Based on the obtained data, the IC_50_ value of porphyrazine was calculated by nonlinear regression using the four-parameter dose-effect model in GraphPad Prism 6 (GraphPad Software).

***Penetration of porphyrazine into SKOV-3 cells.*** To study the distribution of porphyrazine in cells of the monolayer culture, SKOV-3 cells were plated on glass-bottom Petri dishes (Eppendorf, Germany) at 200,000 cells per dish. In 24 h, the growth medium was replaced with serum-free McCoy’s 5A medium containing porphyrazine at concentrations of 1 or 10 μM; the cells were incubated for 4 h at 37°C and 5% CO_2_. Spheroids were treated the same way and then incubated for 1, 4 or 24 h. The incubation periods were chosen based on preliminary results showing that *in vivo* porphyrazine was almost completely eliminated from the tumor 24 h after its intravenous administration.

After incubation, the cells were washed with PBS, fixed with 4% formaldehyde in PBS for 15 min in the dark, and then washed twice with PBS. Images of cells in monolayer and spheroids were obtained using an Axio Observer Z1 LSM 710 NLO Duo confocal microscope (Carl Zeiss, Germany) with C-Apochromat 63×/1·20 W Korr M27 and EC Plan-Neofluar 20×/0.50 M27 lenses, respectively. To excite the fluorescence of porphyrazine, a laser with λ=594 nm was used, and fluorescence was recorded at 600–693 nm.

## Results

***Porphyrazine photodynamic effect on human ovarian adenocarcinoma cells.*** The porphyrazine compound studied in this work is a photosensitizer with a wide absorption band in the red spectral region and fluorescence with the emission maximum at 670 nm [24]. The high lipophilicity of this compound ensures its entry and distribution in the plasma membrane and intracellular membranes.

**Figure 1:**
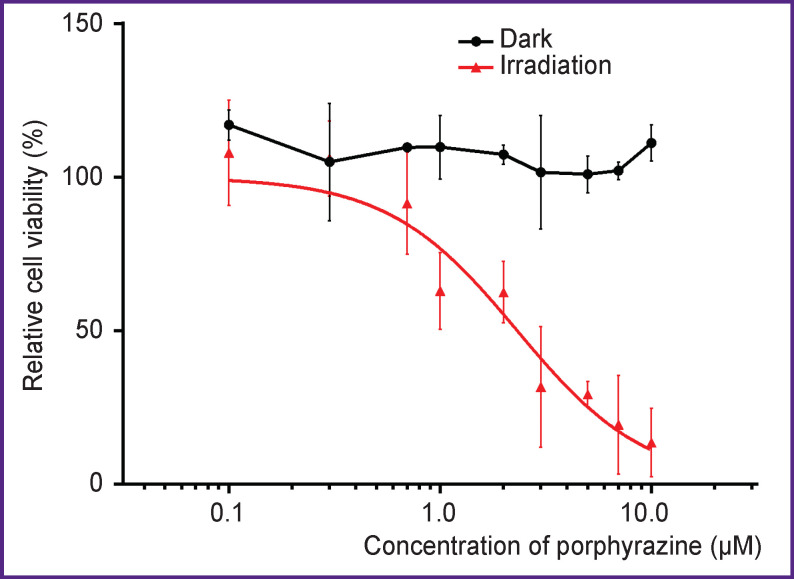
Relative viability of SKOV-3 cells cultured in a monolayer at varying concentrations of porphyrazine in the dark and 24 h after irradiation with light of 615–635 nm at a dose of 20 J/cm^2^ Mean values ± standard error of the mean are shown (n=3)

To analyze its cytotoxicity for the monolayer culture of SKOV-3 cells, the standard MTT assay was used. We found a significant decrease in cell viability resulted from photodynamic effect of porphyrazine at concentrations of 0.1–10.0 μM at an irradiation dose of 20 J/cm^2^; 24 h after irradiation, the IC_50_ value was calculated as 2.3 μM (Figure 1, *red curve*). Notably, in the dark, no toxicity of this compound was observed (Figure 1, *black curve*). Earlier [27], it was shown that for A-431 human epidermoid carcinoma cells, the IC_50_ value of porphyrazine under similar irradiation conditions was significantly lower: 0.68 μM. The difference may be due to a relatively high resistance of SKOV-3 cells to this photosensitizer.

***Photodynamic effect of porphyrazine on spheroids of human ovarian adenocarcinoma.*** To obtain SKOV-3 spheroids, we used round-bottom plates with ultra-low attachment. On day 3 after the cells were plated, they formed spheroids shaped as dense rounded conglomerates with a clearly defined border.

**Figure 2:**
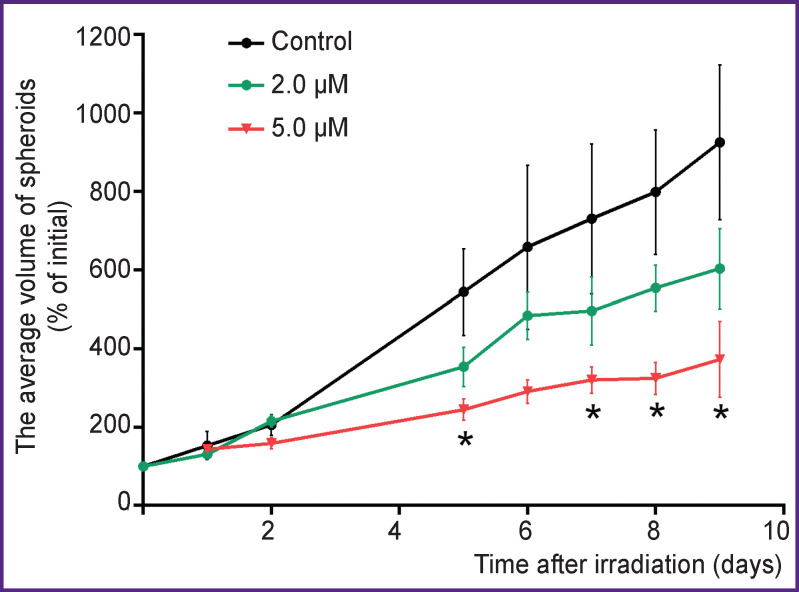
Growth of spheroids without irradiation (control) and after irradiation in the presence of 2.0 or 5.0 μM porphyrazine Data are presented as mean ± standard error of the mean; * statistically significant difference from the control; p<0.05, Dunnett’s test (n=4–6)

The efficacy of the photodynamic action of porphyrazine on spheroids was assessed by their growth rate. In the absence of photodynamic treatment, the relationship close to linear between the spheroid size and the time of incubation was observed for 9 days after their formation (Figure 2). The deviation from the exponential growth typical of cell cultures can be explained by the limited transport of nutrients and gases into the spheroid, as well as by the limited outflow of catabolites; these factors cause a slowdown in cell growth and division and increase the proportion of resting cells instead [17, 28].

**Figure 3:**
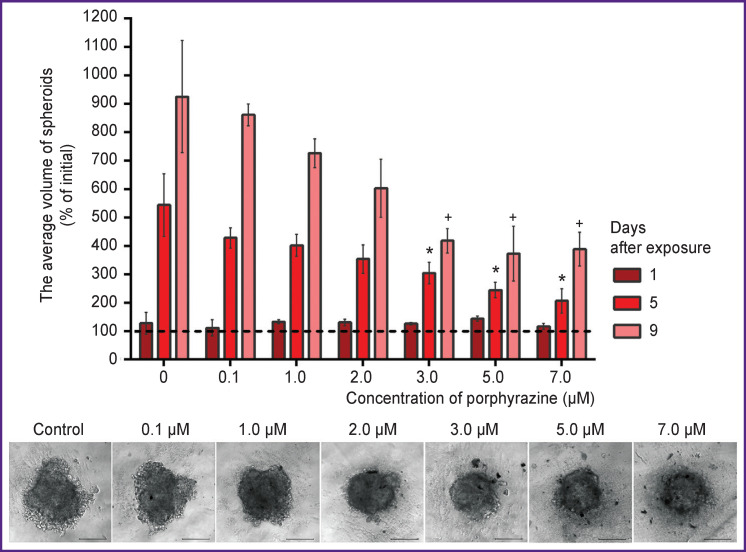
The relative volume of SKOV-3 spheroids at varying concentrations of porphyrazine on days 1, 5, and 9 after irradiation with light of 615–635 nm at a dose of 20 J/cm^2^ The numbers represent the mean values ± standard error of the mean (*above*)*.* Morphology of spheroids at various concentrations of porphyrazine on day 9 after exposure is shown; bar — 200 μm (*below*). *, ^+^ Statistically significant difference from the control (0 μM) on days 5 and 9, respectively (p<0.05, Dunnett test, n=4–6); *dashed line* — initial volume of spheroids (on the day of irradiation)

During the treatment with porphyrazine and subsequent irradiation (20 J/cm^2^), a dose-dependent inhibition of spheroid growth was observed (for the sake of simplicity, the graphs in Figure 2 illustrate only two concentrations of porphyrazine — 2.0 and 5.0 μM). In contrast to the monolayer culture, the effect of porphyrazine on spheroids manifested after some latent period. Thus, no change in the growth rate was detected 24 h after the irradiation (Figure 3, *burgundy bars*), while on days 5 and 9 of incubation, there were significant differences in size between the treated and non-treated spheroids (Figure 3, *red and pink bars*). Microphotographs of spheroids taken on day 9 after photodynamic treatment also show that at high concentrations of porphyrazine, spheroids loosen, lose their clear outline and become surrounded by debris, which is most likely due to cell death in the outer layers of the spheroid.

It should be noted that under none of the regimes of photodynamic exposure we observed a decrease in the initial size of the treated spheroids. It can be assumed that even after irradiation, a substantial population of viable cells exists in the spheroids and it ensures continued survival of the spheroid. Thus, Figure 3 shows that the spheroid growth was at least 40% of control, even under conditions leading to almost complete cell death in a monolayer culture. At the same time, the calculated IC_50_ values on days 5 and 9 of incubation were 3.5 and 1.8 μM, respectively, which is close to the IC_50_ value for the cell monolayer 24 h after irradiation.

The relationship between the 3D structure of spheroids and their resistance to photodynamic damage is ambiguous. According to some reports, no significant differences were found between spheroids and monolayer cultures in their sensitivity to photodynamic damage in the presence of a ruthenium-based hybrid nanophotosensitizer [29] or organic nanoparticles containing chlorin e6 [30]. Most researchers, however, hold a point of view that tumor cells are more resistant to photodynamic effects when they are cultured as a 3D model [22, 31, 32]. As possible reasons for high resistance, the low photosensitizer penetration into cell mass is often considered. Among other mechanisms, there are hypoxic conditions caused by the rapid consumption of oxygen during the photodynamic reaction, increased expression of proteins — ABC transporters that actively pump the chromophore out of tumor cells [22], as well as the recently shown inhibition of genes of several pro-apoptotic proteins [21].

***Accumulation of porphyrazine in human ovarian adenocarcinoma cells in a monolayer culture and in spheroids.*** In a search for a possible mechanism behind the resistance of spheroids to photodynamic treatment, we analyzed the accumulation of porphyrazine in SKOV-3 cells cultured as a monolayer or as 3D spheroids.

**Figure 4:**
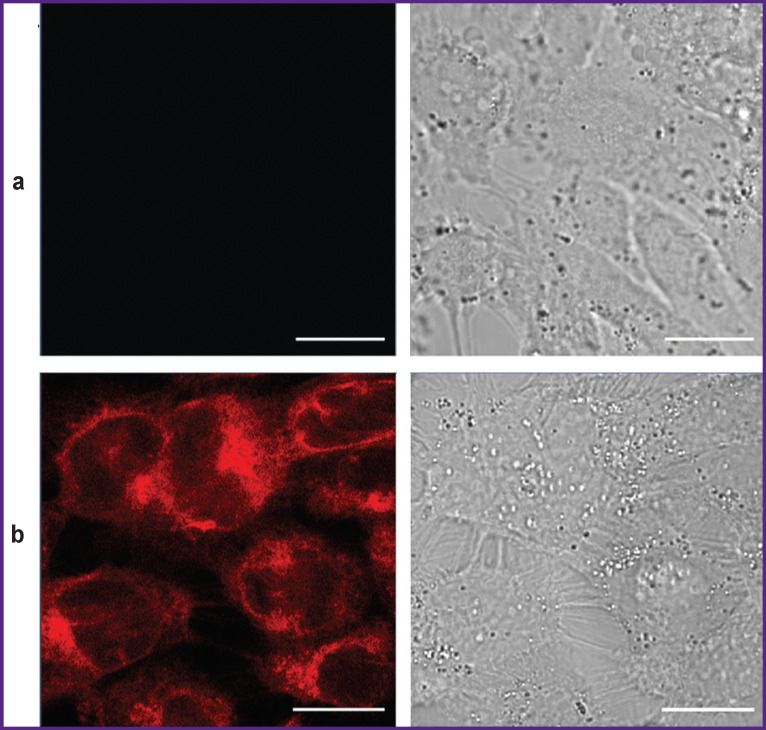
Accumulation of porphyrazine in SKOV-3 cells in a monolayer: confocal microscopy of cells (a) in control (in the absence of porphyrazine); (b) after 4 h of incubation in the presence of 1.0 μM porphyrazine; images were obtained in the porphyrazine fluorescence channel (*left*) and in transmitted light (*right*); bar — 20 μm

Using confocal microscopy, we found that over the course of 4 h, porphyrazine intensively accumulated inside SKOV-3 cells in the monolayer culture, where it stained intracellular membranes, presumably, the Golgi apparatus and endoplasmic reticulum (Figure 4).

**Figure 5:**
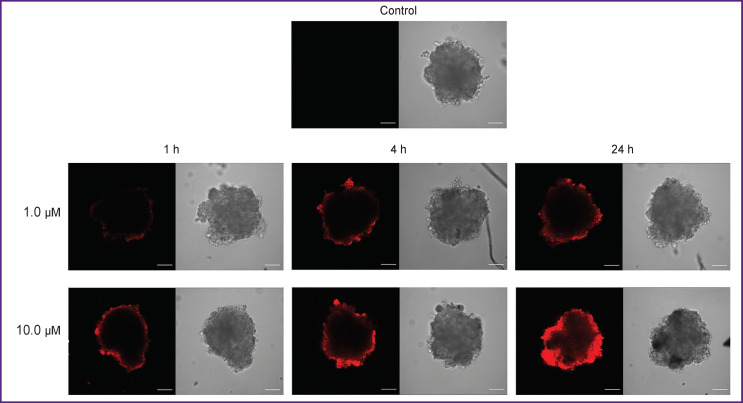
Accumulation of porphyrazine in SKOV-3 spheroids: confocal microscopy of spheroids in control (in the absence of porphyrazine) and after 1, 4 or 24 h of incubation in the presence of 1.0 or 10.0 μM porphyrazine; images were obtained in the porphyrazine fluorescence channel (*left*) and in transmitted light (*right*); bar — 100 μm

In spheroids, this process developed much slower (Figure 5). During the first few hours, only the surface cell layers demonstrated the detectable level of fluorescence. After 24 h incubation in the presence of 1.0 μM porphyrazine, the dye penetrated to a depth of ~50 μm into a spheroid of 400–450 μm in diameter. At a concentration of 10.0 μM of porphyrazine, a more intense fluorescence was detected in all parts of the spheroid; however, even in this case, the bulk of the fluorescent signal was recorded at depths not exceeding 100 μm.

Most of the currently used photosensitizers have molecular weights of 1–2 kDa. A large number of cell-cell and cell-matrix contacts in the 3D structure of tumors restrict diffusion of compounds having medium and high molecular weights; such compounds do not penetrate into the tumor tissue deeper than 100–150 μm [33, 34]. These penetration depths were determined in 3D *in vitro* tumor models exposed to various photosensitizers [32, 35]. According to our data, the accumulation of porphyrazine mainly in the surface cell layers can explain the significantly different photodynamic effects on the surface as compared to the deep layers of the spheroid. For cells located close to the surface, the photoinduced toxicity of porphyrazine is comparable to that for a monolayer culture, resulting in comparable IC_50_ values. The deeper layers with low accumulation of the photosensitizer are characterized by low sensitivity to irradiation. The cells in these depths remain viable and provide continued growth of the spheroid even after an intense exposure.

The limited penetration of photosensitizers from the blood into the tumor tissue *in vivo* is the reason for their inhomogeneous distribution, which ultimately reduces the efficacy of photodynamic therapy. It should be kept in mind that intravenously administered photosensitizers form complexes with blood plasma proteins, mainly with albumin and lipoproteins, and in this form are transported through the circulation [36]. Extravasation of photosensitizers, their diffusion through the extracellular matrix, and interaction with cells also occur by virtue of their complexes with proteins, which may slow the distribution even more. One of the proposed approaches to solving the problem is to use vehicles capable to increase the tissue penetration of photodynamic agents. Liposomes, organic and hybrid nanoparticles, membrane-based vesicles have been tried for this role [27, 32, 35, 37]. The advantages of such carriers are expected to increase the efficacy of photodynamic therapy.

## Conclusion

Response of ovarian adenocarcinoma cells to photodynamic treatment mediated by a photosensitizer from the tetra(aryl)tetracyanoporphyrazines group depends on the conditions of cell cultivation. In the case of tumor spheroids, their three-dimensional structure ensures the preservation of partial cell viability and continuing growth of spheroids even under intensive photodynamic exposure. The high photo-resistance of these cells is mainly due to the limited penetration of the photosensitizer into the spheroid, not exceeding 100–150 μm. The *in vitro* distribution of photosensitizers in the model spheroids is similar to their inhomogeneous accumulation in tumors *in vivo*; therefore, we consider spheroids as a model more relevant to this kind of research than a cell monolayer. Using tumor spheroids and other 3D models of tumor growth *in vitro* will allow us to better assess the therapeutic efficacy of potential drugs and provide a tool for testing approaches to improve PDT efficacy.

## References

[1] Triesscheijn M., Baas P., Schellens J.H.M., Stewart F.A (2006). Photodynamic therapy in oncology.. Oncologist.

[2] Hamblin M.R., Mroz P (2008). History of PDT: the first hundred years.. Advances in photodynamic therapy: basic, translational and clinical..

[3] Robertson C.A., Evans D.H., Abrahamse H (2009). Photodynamic therapy (PDT): a short review on cellular mechanisms and cancer research applications for PDT.. J Photochem Photobiol B.

[4] Krasnovsky A.A (2008). Luminescence and photochemical studies of singlet oxygen photonics.. J Photochem Photobiol A Chem.

[5] Brilkina A.A., Peskova N.N., Dudenkova V.V., Gorokhova A.A., Sokolova E.A., Balalaeva I.V (2018). Monitoring of hydrogen peroxide production under photodynamic treatment using protein sensor HyPer.. J Photochem Photobiol B.

[6] Choi Y., Chang J.E., Jheon S., Han S.J., Kim J.K (2018). Enhanced production of reactive oxygen species in HeLa cells under concurrent low-dose carboplatin and Photofrin® photodynamic therapy. Oncol Rep.

[7] Abrahamse H., Hamblin M.R (2016). New photosensitizers for photodynamic therapy.. Biochem J.

[8] Garland M.J., Cassidy C.M., Woolfson D., Donnelly R.F (2009). Designing photosensitizers for photodynamic therapy: strategies, challenges and promising developments.. Future Med Chem.

[9] Ethirajan M., Chen Y., Joshi P., Pandey R.K (2011). The role of porphyrin chemistry in tumor imaging and photodynamic therapy.. Chem Soc Rev.

[10] Ormond A.B., Freeman H.S (2013). Dye sensitizers for photodynamic therapy.. Materials.

[11] Chilakamarthi U., Giribabu L (2017). Photodynamic therapy: past, present and future.. Chem Rec.

[12] Brilkina A.A., Dubasova L.V., Sergeeva E.A., Pospelov A.J., Shilyagina N.Y., Shakhova N.M., Balalaeva I.V (2019). Photobiological properties of phthalocyanine photosensitizers Photosens, Holosens and Phthalosens: a comparative in vitro analysis.. J Photochem Photobiol B.

[13] Shilyagina N.Y., Peskova N.N., Lermontova S.A., Brilkina A.A., Vodeneev V.A., Yakimansky A.V., Klapshina L.G., Balalaeva I.V (2017). Effective delivery of porphyrazine photosensitizers to cancer cells by polymer brush nanocontainers.. J Biophotonics.

[14] Lermontova S.A., Grigor’ev I.S., Ladilina E.Yu., Balalaeva I.V., Shilyagina N.Yu., Klapshina L.G. (2018). Porphyrazine structures with acceptor substituents as the basis of materials for photonics and biomedicine.. Koordinatsionnaya khimiya.

[15] Haidekker M.A., Theodorakis E.A (2010). Environment-sensitive behavior of fluorescent molecular rotors.. J Biol Eng.

[16] Izquierdo M.A., Vyšniauskas A., Lermontova S.A., Grigoryev I.S., Shilyagina N.Y., Balalaeva I.V., Klapshina L.G., Kuimova M.K (2015). Dual use of porphyrazines as sensitizers and viscosity markers during photodynamic therapy.. J Mater Chem B.

[17] Thoma C.R., Zimmermann M., Agarkova I., Kelm J.M., Krek W (2014). 3D cell culture systems modeling tumor growth determinants in cancer target discovery.. Adv Drug Deliv Rev.

[18] Unger E., Porter T., Lindner J., Grayburn P. (2014). Cardiovascular drug delivery with ultrasound and microbubbles.. Adv Drug Deliv Rev.

[19] Sokolova E.A., Vodeneev V.A., Deyev S.M., Balalaeva I.V (2019). 3D in vitro models of tumors expressing EGFR family receptors: a potent tool for studying receptor biology and targeted drug development.. Drug Discov Today.

[20] Mohammad-Hadi L., MacRobert A.J., Loizidou M., Yaghini E. (2018). Photodynamic therapy in 3D cancer models and the utilisation of nanodelivery systems.. Nanoscale.

[21] Manoto S.L., Houreld N., Hodgkinson N., Abrahamse H (2017). Modes of cell death induced by photodynamic therapy using zinc phthalocyanine in lung cancer cells grown as a monolayer and three-dimensional multicellular spheroids.. Molecules.

[22] Khot M.I., Perry S.L., Maisey T., Armstrong G., Andrew H., Hughes T.A., Kapur N., Jayne D.G (2018). Inhibiting ABCG2 could potentially enhance the efficacy of hypericin-mediated photodynamic therapy in spheroidal cell models of colorectal cancer.. Photodiagnosis Photodyn Ther.

[23] Balalaeva I.V., Sokolova E.A., Puzhikhina A.D., Brilkina A.A., Deyev S.M (2017). Spheroids of HER2-positive breast adenocarcinoma for studying anticancer immunotoxins in vitro.. Acta Naturae.

[24] Lermontova S.A., Grigor’ev I.S., Ladilina E.Y., Klapshina L.G., Peskova N.N., Balalaeva I.V., Boyarskii V.P (2017). New promising porphyrazine-based agents for optical theranostics of cancer.. Russ J Gen Chem.

[25] Shilyagina N.Y., Plekhanov V.I., Shkunov I.V., Shilyagin P.А., Dubasova L.V., Brilkina А.А., Sokolova E.A., Turchin I.V., Balalaeva I.V. (2014). LED light source for in vitro study of photosensitizing agents for photodynamic therapy.. Sovremennye tehnologii v medicine.

[26] Mosmann T (1983). Rapid colorimetric assay for cellular growth and survival: application to proliferation and cytotoxicity assays.. J Immunol Methods.

[27] Yudintsev A.V., Shilyagina N.Yu., Dyakova D.V., Lermontova S.A., Klapshina L.G., Guryev E.L., Balalaeva I.V., Vodeneev V.A. (2018). Liposomal form of tetra(aryl) tetracyanoporphyrazine: physical properties and photodynamic activity in vitro.. J Fluoresc.

[28] Huang B.W., Gao J.Q (2018). Application of 3D cultured multicellular spheroid tumor models in tumor-targeted drug delivery system research.. J Control Release.

[29] Zhang D.Y., Zheng Y., Zhang H., He L., Tan C.P., Sun J.H., Zhang W., Peng X., Zhan Q., Ji L.N., Mao Z.W (2017). Ruthenium complex-modified carbon nanodots for lysosome-targeted one- and two-photon imaging and photodynamic therapy.. Nanoscale.

[30] Kumari P., Rompicharla S.V.K., Bhatt H., Ghosh B., Biswas S (2019). Development of chlorin e6-conjugated poly(ethylene glycol)-poly(d,l-lactide) nanoparticles for photodynamic therapy.. Nanomedicine (Lond).

[31] Rizvi I., Celli J.P., Evans C.L., Abu-Yousif A.O., Muzikansky A., Pogue B.W., Finkelstein D., Hasan T (2010). Synergistic enhancement of carboplatin efficacy with photodynamic therapy in a three-dimensional model for micrometastatic ovarian cancer.. Cancer Res.

[32] Millard M., Yakavets I., Piffoux M., Brun A., Gazeau F., Guigner J.M., Jasniewski J., Lassalle H.P., Wilhelm C., Bezdetnaya L (2018). mTHPC-loaded extracellular vesicles outperform liposomal and free mTHPC formulations by an increased stability, drug delivery efficiency and cytotoxic effect in tridimensional model of tumors.. Drug Deliv.

[33] Choi I.K., Strauss R., Richter M., Yun C.O., Lieber A (2013). Strategies to increase drug penetration in solid tumors.. Front Oncol.

[34] Namazi H., Kulish V.V., Wong A., Nazeri S (2016). Mathematical based calculation of drug penetration depth in solid tumors.. Biomed Res Int.

[35] Yakavets I., Lassalle H.P., Scheglmann D., Wiehe A., Zorin V., Bezdetnaya L (2018). Temoporfin-in-cyclodextrin-in-liposome — a new approach for anticancer drug delivery: the optimization of composition.. Nanomaterials (Basel).

[36] Castano A.P., Demidova T.N., Hamblin M.R (2005). Mechanisms in photodynamic therapy: part three — photosensitizer pharmacokinetics, biodistribution, tumor localization and modes of tumor destruction.. Photodiagnosis Photodyn Ther.

[37] Lee J., Kim J., Jeong M., Lee H., Goh U., Kim H., Kim B., Park J.H (2015). Liposome-based engineering of cells to package hydrophobic compounds in membrane vesicles for tumor penetration.. Nano Lett.

